# Exploiting pH-Regulated Dimer-Tetramer Transformation of Concanavalin A to Develop Colorimetric Biosensing of Bacteria

**DOI:** 10.1038/s41598-017-01371-6

**Published:** 2017-05-03

**Authors:** Xiahong Xu, Yuwei Yuan, Guixian Hu, Xiangyun Wang, Peipei Qi, Zhiwei Wang, Qiang Wang, Xinquan Wang, Yingchun Fu, Yanbin Li, Hua Yang

**Affiliations:** 1 State Key Lab Breeding Base for Zhejiang Sustainable Plant Pest Control; Ministry of Agriculture Key Lab for Pesticide Residue Detection; Key Laboratory of Detection for Pesticide Residues and Control of Zhejiang Province, Institute of Quality and Standard for Agro-products, Zhejiang Academy of Agricultural Sciences, Hangzhou, 310021 P.R. China; 2 College of Biosystems Engineering and Food Science, Zhejiang University, Hangzhou, 310058 P.R. China

## Abstract

Gold nanoparticles (AuNPs) aggregation-based colorimetric biosensing remains a challenge for bacteria due to their large size. Here we propose a novel colorimetric biosensor for rapid detection of *Escherichia coli* O157:H7 (*E*. *coli* O157:H7) in milk samples based on pH-regulated transformation of dimer/tetramer of *Concanavalin* A (Con A) and the Con A-glycosyl recognition. Briefly, antibody-modified magnetic nanoparticles was used to capture and concentrate *E*. *coli* O157:H7 and then to label with Con A; pH adjusted to 5 was then applied to dissociate Con A tetramer to release dimer, which was collected and re-formed tetramer at pH of 7 to cause the aggregation of dextran-modified AuNPs. The interesting pH-dependent conformation-transformation behavior of Con A innovated the design of the release from the bacteria surface and then the reconstruction of Con A. Therefore, we realized the sensitive colorimetric biosensing of bacteria, which are much larger than AuNPs that is generally not suitable for this kind of method. The proposed biosensor exhibited a limit of detection down to 41 CFU/mL, short assay time (~95 min) and satisfactory specificity. The biosensor also worked well for the detection in milk sample, and may provide a universal concept for the design of colorimetric biosensors for bacteria and virus.

## Introduction

The World Health Organization (WHO) has estimated 600 million (almost 1 in 10 people in the world) fall ill after eating contaminated food and 420 000 die every year, resulting in the loss of 33 million healthy life years (DALYs)^1^. *Escherichia coli* O157:H7 (*E*. *Coli* O157:H7) is an enterohaemorrhagic, verocytotoxin-producing, or shiga-toxin-producing pathogen, which has been identified as a global threat associated with human health and food safety. The presence of *E*. *Coli* O157:H7 in food is not only from animal-sourced food stock (such as unpasteurized milk, undercooked meat) but also from vegetables (such as fresh fruits and vegetables) grown in infested soil and water. Thus it is important to obtain a rapid, sensitive, and specific technique for *E*. *Coli* O157:H7 detection. Several techniques are available in laboratories for pathogenic bacteria detection and identification, including plating and culturing^2^, surface plasmon resonance^3–6^, electrochemistry^7–13^, quartz crystal microbalance^14–16^, fluorescent methods^17–22^. Each of these systems has its advantages, however, the utility of these methods is generally limited by their high cost and the requirement of trained operators.

Recent advances in colorimetric biosensors have shown great potential to satisfy the practical need for rapid detection with features of sensitive and rapid readout, portability and cost-efficiency^23^. Functionalized gold nanoparticles (AuNPs)-based colorimetric biosensors have drawn increased attention^24–34^. The reduction of the distance between AuNPs (d > 3.5 nm) induces inter-particle surface plasmon coupling, resulting in a visible color change from red to blue at nanomolar concentrations. This corresponding color change of AuNPs as an analytical signal is easy to realize for the detection of small size analytes, such as metal ions, anions, small organic molecules, DNA and protein, due to the interaction in nano-scale between analytes and AuNPs^34^. However, it is difficult to develop AuNPs aggregation-based colorimetric biosensors for large dimension analytes, especially for bacteria. Because the AuNPs-based colorimetric method relies on the gap between two inidividual Au particle which should be less than 10 nm. It is easy for the small molecule generally with size of nanometer/sub-nanmeter scale but is not possible for bacteria with micrometer size. Instead, AuNPs tend to adsorb on the surface of the bacteria and have minor color change. Therefore, current colorimetric biosensing methods of bacteria are generally based on the aggregation of AuNPs induced by species released from bacteria (DNA, protein, etc.) or some catalytic labels (enzyme, nanoparticles, etc.)^9^. However, these indirect methods requires complicated pretreatment of bacteria or/and preparation of labelling complexes. Consequently, the development of AuNPs aggregation-based colorimetric biosensor with high sensitivity and simplicity for bacteria is necessary but still remains a great challenge.

Lectin of *Concanavalin* A (Con A) can strongly bind to specific glycosyl moieties on the surface of bacteria. Lectins as molecular recognition elements or bioconjugation materials for functionalizing nanoparticles are particularly interesting because of their easy production and intrinsic stability^5, 8^. Therefore, Con A has been broadly selected as the capturing probe and labeling tool. On the other hand, Con A also exhibits interesting dimer-tetramer equilibrium in the pH range 5–6^35^, namely, Con A could dissociate into dimeric and monomeric subunits under pH 5, and reconstruct as tetramer at pH higher than 6. This interesting dissociation-reconstruction behavior is similar to the AuNPs-based dispersion-aggregation, which may inspire a novel colorimetric strategy for bacteria taking consideration of the strong recognition ability of Con A to bacteria.

**Figure 1:**
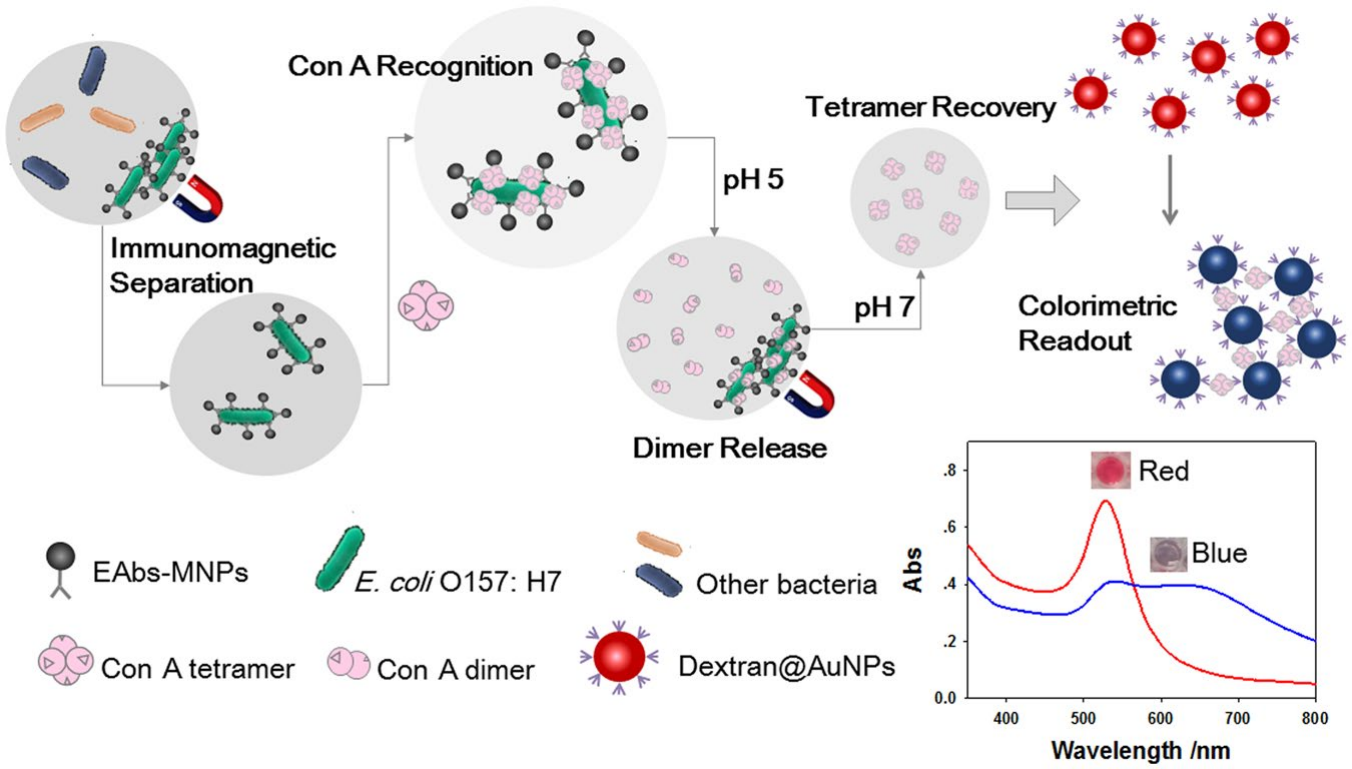
Illustration of the procedures and mechanism of the colorimetric biosensor.

In this study, we described a novel colorimetric biosensor for the detection of pathogens based on pH-regulated transformation of dimer/tetramer of Con Aand the Con A-glycosyl recognition. As shown in Fig. 1, antibody-modified magnetic nanoparticles was used to capture and concentrate the target *E*. *coli* O157:H7 and then to label the bacteria with Con A. By regulating pH to below 5, tetramer Con A dissociated into dimer isoforms and released from bacterial cells surface, and then the acidity was adjusted back to pH 7 to reconstruct tetramer Con A, which readily caused the aggragation of dextran@AuNPs, thus resulting in color change from red to blue by AuNPs aggregation, and providing a colorimetric readout. This method takes advantages of the interesting dimer-tetramer transformation of Con A by facile pH-regulation. The high abundance of binding sites of bacteria for Con A, and the strong ability of Con A to induce aggregation of AuNPs, provides a sensitive and simple detection platform for bacteria.

## Experimental Section

### Materials and reagents

The biotin-conjugated rabbit antibody to *E*. *coli* O157:H7, O and K antigenic serotypes, was purchased from Meridian Life Science, Inc. (Memphis, USA). The carboxyl conjugated magnetic nanoparticles (MNPs) were purchased from Allrun Nano (PM3-020, Shanghai, China). Gold chloride (HAuCl_4_ · 3H_2_O), streptavidin, 1-(3-Dimethylaminopropyl)-3-ethylcarbodiimide hydrochloride (EDC·HCl), N-Hydroxysuccinimide (NHS), phosphate buffered saline (PBS, pH 7.4, diluted to 10 mM before use), Con A from Canavalia ensiformis (Jack bean, Type IV, lyophilized powder) were purchased from Sigma-Aldrich (St. Louis, MO, US). Bovine serum albumin (BSA) from EM Science (Gibbstown, NJ, US) was prepared in PBS for blocking. Dextran (40,000) was purchased from BBI (Sangon Biotech., Shanghai, China). Tween-20 was purchased from Amersco (Solon, OH, US) for washing. Other reagents were of analytical grade. Ultra-purified water produced by Advantage A10 from Millipore (Billerica, MA, USA) was used throughout.

### Preparation of dextran-capped AuNPs

Dextran-capped AuNPs were prepared at room temperature (25 °C) using dextran as both a reducing agent and a stabilizer according to published method^36^. Firstly, 47.0 mL of Milli-Q water, 10.0 mg of dextran, and 2.0 mL of 1.0% (w/w) HAuCl_4_ were mixed in a flask with ultrasonic irradiation. Then, 1.0 mL of 1.0 M NaOH was quickly added to the solution under vigorous magnetic stirring to adjust the pH to ca. 11, and the color of the solution changed to deep red within 16 h.

### Preparation and culture of bacteria

*E*. *coli* O157:H7 (ATCC43889) used as target bacteria and *L*. *monocyto genes* (ATCC19114) and *S*. *Typhymurium* (ATCC 14028) were used as non-target bacteria which were stored at −20 °C with 15% glycerol and were revived by streaking on Luria-Bertani (LB) agar plates. They were first cultured in LB medium (Aoboxing Biotech, Beijing, China) at 37 °C for 12–16 h with shaking at 180 rpm. Then, the cultures were 10-fold diluted with sterile PBS to obtain the bacteria at the concentrations ranging from 10^1^ to 10^4^ CFU/mL. For bacterial enumeration, the bacterial samples were serially diluted with sterile PBS and 100 μL of the diluents were plated on the LB agar plates. The plates were then incubated at 37 °C for 22–24 h before the colonies were counted. The bacteria were enumerated in colony forming unit per milliliter (CFU/mL).

### Modification of MNPs

The monodispersed MNPs with a diameter of 180 nm were functionalized with carboxyl group (~150 μM/g) and stored in water at room temperature. The core of the MNPs is Fe_3_O_4_ with the Fe content of 10 mg/mL. The anti-*E*. *coli* O157:H7 monoclonal antibodies (EAbs) were immobilized on the surface of MNPs by the reported protocol with minor modifications^37^. Prior to use, 2 mg of the carboxylated MNPs were successively washed with 1 M HCl and water using a magnetic separator with a max magnetic strength of ~1.1 T from Aibit Biotech (MS0206, Jiangyin, China). First, EDC and NHS (2 mg in 500 μL of water) were freshly prepared and used to activate the carboxyl groups at room temperature for 30 min. After washing with deionized water twice to remove surplus EDC and NHS, the MNPs were immediately mixed with 400 mL of PBS and 100 μL of streptavidin (1 mg/mL in PBS), and gently rotated at 15 rpm for 1 h. After washing with PBS twice, the streptavidin modified MNPs were incubated with 1% BSA for 45 min to block the non-specific sites. After washing with PBS for three times, the BSA-blocked MNPs were re-suspended in 1.5 mL of PBS containing 1.33 mg EAbs (~1.15 mg/mL) and incubated for 45 min with rotating at 15 rpm. After washing with PBS twice, the EAbs modified MNPs were finally suspended in PBS containing 1% BSA at a final concentration of 1 mg/mL (Fe content) and stored at 4 °C.

### Separation efficiency of EAbs modified MNPs against *E. coli* O157:H7

Serial dilutions of the pure cultures of *E*. *coli* O157:H7, were prepared in PBS as mentioned above. EAbs-modified MNPs of 20 μL was mixed with 200 μL culture (10^3^, 10^4^ and 10^5^ CFU/mL) and rotated at 15 rpm for 45 min at RT. The MNPs-bacteria complexes were washed two times and re-suspended in 200 μL of PBS. The uncaptured cells in the supernatant were also collected and appropriately diluted if needed. 100 μL of the captured samples and uncaptured samples were plated on selective agars and incubated at 37 °C for 24 h for bacterial enumeration. The same level for all the original cultures was used as a positive control. All enumeration experiments were performed in triplicate.

### Colorimetric detection of *E*. *coli* O157:H7 based on pH-regulation

One hundred and fifty microlitres of the EAbs modified MNPs suspension was incubated with 200 μL of bacteria solution with concentrations ranging from 10^2^ to 10^9^ CFU/mL for 45 min in a 2 mL sterile centrifuge tube with rotating at 15 rpm (the tube was pre-blocked using 1% BSA for 30 min prior to use). After washing with 0.5 mL PBS twice (for 5 min), the bacteria-bound MNPs were resuspended in 200 μL PBS reaction solution (0.01 M, pH 6.8, containing 0.1 mM Ca^2+^, 0.1 mM Mn^2+^) containing 0.5 mg/mL Con A, followed by incubation at 15 rpm for 30 min to label Con A onto the surface of the bacteria. The mixture reaction solutions were sequentially washed with 200 μL washing solution (10 mM PBS, pH 7.4, 0.05% Tween 20, PBST) once and water for three times to remove the unbound Con A. Then bacteria solutions were resuspended in 50 μL PBS (0.01 M, pH 5) to release Con A dimer, followed by transporting supernatants to new tubes and adjusting pH back to 7 by NaOH (termed as Final-solution), this process of changing pH of the solution to obtain the F-solution needs about 10 min. Finally, these F-solutions were mixed with 50 μL dextran@AuNPs for about 5 min, absorption spectra were collected using UV-vis spectrophotometer.

For specificity of detection, 150 μL of the EAbs-modified MNPs suspension was incubated with 200 μL of 1.01 × 10^7^ CFU/mL *E*. *coli* O157:H7, 1.03 × 10^7^ CFU/mL *S*. *Typhimurium*, 1.04 × 10^7^ CFU/mL *L*. *monocytogenes*, and a complex bacteria solution with those three pathogens, respectively, for 45 min in a 2 mL sterile centrifuge tube rotating at 15 rpm (the tube was pre-blocked using 1% BSA for 30 min prior to use). The other steps were the same as described above for colorimetric detection of *E*. *coli* O157:H7.

## Results and Discussion

**Figure 2:**
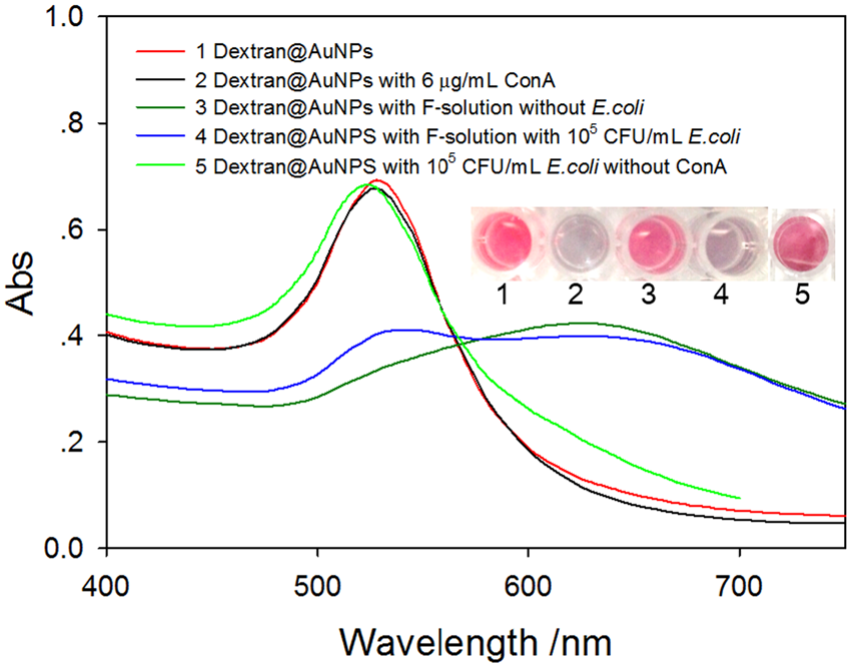
UV-vis spectra of dextran@AuNPs before (curve 1) and after (curve 2) mixing with Con A in PBS, pH 7.4, or mixed with Final-solution without bacteria (curve 3) or with 10^5^ CFU/mL *E*. *coli* O157:H7 (curve 4), or mixed with 10^5^ CFU/mL *E*. *coli* O157:H7 but without ConA (curve 5).

Since Con A-induced aggregation of AuNPs is the basis for final signal readout, the ability of Con A for this purpose was evaluated first using UV-vis spectrophotometry. The UV-vis spectrum of dextran@AuNPs showed a strong absorption peak at 525 nm (as shown in Fig. 2, curve 1). After dextran@AuNPs were mixed with 6 µg/mL Con A, the color of AuNPs immediately changed from red to blue, because dextran@AuNPs were crosslinked by Con A and in turn aggregated from scattered state (curve 2), which resulted in the attenuation of the absorption peak at 525 nm as well as the appearance of a new peak at ca. 635 nm. After mixing of the dextran@AuNPs with the Final-solution for 1.02 × 10^5^ CFU/mL *E*. *coli* O157:H7, similar phenomenon as that of the sample 2 was observed. As shown in Fig. 2, curve 4, greatly reduced absorption peak at 525 nm and the appearance of the new peak at ca. 635 nm were found according to the UV-vis spectrum curve. Comparing with curve 2, Curve 4 showed an absorption peak at 525 nm. The reason might be that tetramer ConA released from the surface of *E*. *coli* was not able to gather all AuNPs at the concentration of *E*. *coli* (10^5^ CFU/mL), resulting in a part of the AuNPs remain dispersed and the presence of a small absorption peak at 525 nm. In contrast, the mixing of dextran@AuNPs with Final-solution without bacteria presented minor change as the primary dextran@AuNPs (curve 3). Moreover, the mixing of dextran@AuNPs with 10^5^ CFU/mL *E*. *coli* in the absence of ConA presented minor color change of AuNPs as well as minor change on the absorption peak at 525 nm (curve 5). Above results indicate that Con A released from the surface of bacteria could readily induce the aggregation of AuNPs for signal readout rather than bacteria themselves did.

**Figure 3:**
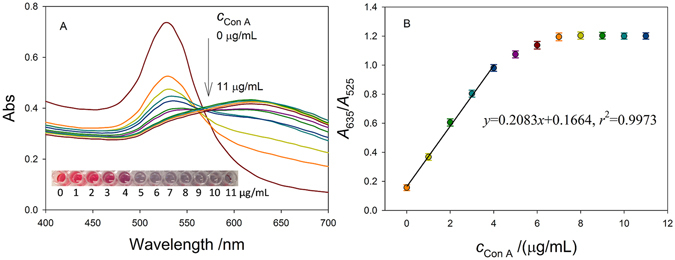
(**A**) UV-vis spectra of dextran@AuNPs mixed with different concentration of Con A, 0, 1, 2, 3, 4, 5, 6, 7, 8, 9, 10, 11 μg/mL. (**B**) The absorption ratio in 635 nm and 525 nm for the concentration of Con A.

To evaluate the efficiency that Con A induce the aggregation of dextran@AuNPs, we collected UV-vis spectra curves of the solutions of dextran@AuNPs mixing with different concentrations of Con A. As shown in Fig. 3A, with the increase in concentration of Con A, absorption intensity at 525 nm and 635 nm showed gradual decrease and increase, respectively, indicating gradual aggregation of the dextran@AuNPs. To understand the effect of the concentration of Con A, we further examined the trend of the adsorption ratio of 525 nm to 635 nm along with the concentration of Con A. We found a good linear range from 1 to 4 μg/mL (*R* ^2^ = 0.9973) and a detection limit of 0.24 μg/mL. Considering the volume of 10 μL and the molecular weight of 102000 g mol^−1^ of Con A, we calculated the amount of Con A is 1.416 × 10^10^. Taking into account that there are about 3.6 × 10^6^ lipopolysaccharides (LPS) on the surface of one bacterial cell and each LPS has a basic O-antigen chain length of 10 to 18 O units with repeating units of 1 to >100 polysaccharide (averaging 10)^38–40^, we evaluated that potentially from 2 to 393 bacteria (1.416 × 10^10^/(18 × 100 × 3.6 × 10^6^) to 1.416 × 10^10^/(10 × 1 × 3.6 × 10^6^)) might be enough to cause a detectable change based on the aggregation of AuNPs. The recovery rate during/after the dimer release ConA was also calculated to be 89.7% in our testing system (details in Figure S1). These results demonstrat the high sensitivity of the proposed method.

The separation efficiency (SE) of *E*. *coli* O157:H7 using the EAbs modified MNPs directly affects the sensitivity of this biosensor. SE was defined as the percentage of the magnetically separated bacteria compared to the total bacteria in the positive control and calculated as:

$${\rm{S}}{\rm{E}}({\rm{ \% }})={N}_{{\rm{m}}{\rm{s}}}/{N}_{{\rm{p}}{\rm{c}}}\times 100{\rm{ \% }}$$

where *N*_ms_ is the number of the magnetically separated bacteria cells and *N*_pc_ is the number of the positive control. Three parallel tests were conducted at concentrations of *E*. *coli* O157:H7 ranging from 10^2^ to 10^4^ CFU/mL in pure cultures. The SEs of *E*. *coli* at concentrations were all larger than 95% in the pure cultures (details in Table S1).

**Figure 4:**
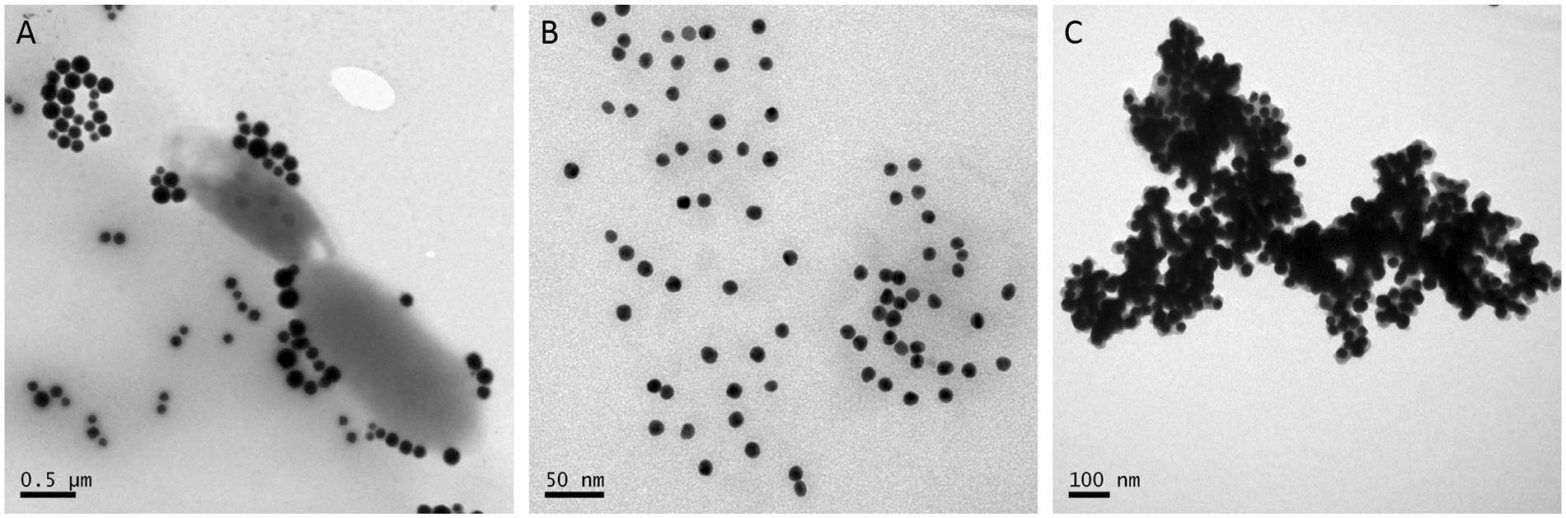
TEM imaging of EAbs modified MNPs coupled into bacterial cell surface (**A**); dextran@AuNPs before (**B**) and after (**C**) coupled with released Con A in the Final-solution.

TEM imaging was used to further investigate the magnetic separation and Con A-induced aggregation of AuNPs. As shown in Fig. 4A, after the capturing and magnetic separation, we found that bacterial cells were surrounded by EAbs-modified MNPs with the number generally larger than ten per cell, which agreed well with that of 95% of SE. For the dextran@AuNPs, we observed mono-dispersed particles with average diameter of 15 nm (Fig. 4B). After the mixing of dextran@AuNPs with the Final-solution, obvious aggregation of dextran@AuNPs was observed (Fig. 4C), proving again the high efficiency of the coupling of dextran@AuNPs by Con A, as well as that the pH-regulation was efficient in releasing and reconstructing of Con A dimers. More TEM pictures are shown in Figure S2. As a control, dextran@AuNPs aggregated in the presence of ConA (Figure S2A). While the F-solution without ConA did not result in aggregation (Figure S2C), the results may prove that only the change of pH for F-solution is not enough to make dextran@AuNPs aggregation, and this interference to our sensing system can be ignored. Therefore, the proposed method should be reliable for colorimetric detection of *E*. *coli* O157:H7.

**Figure 5:**
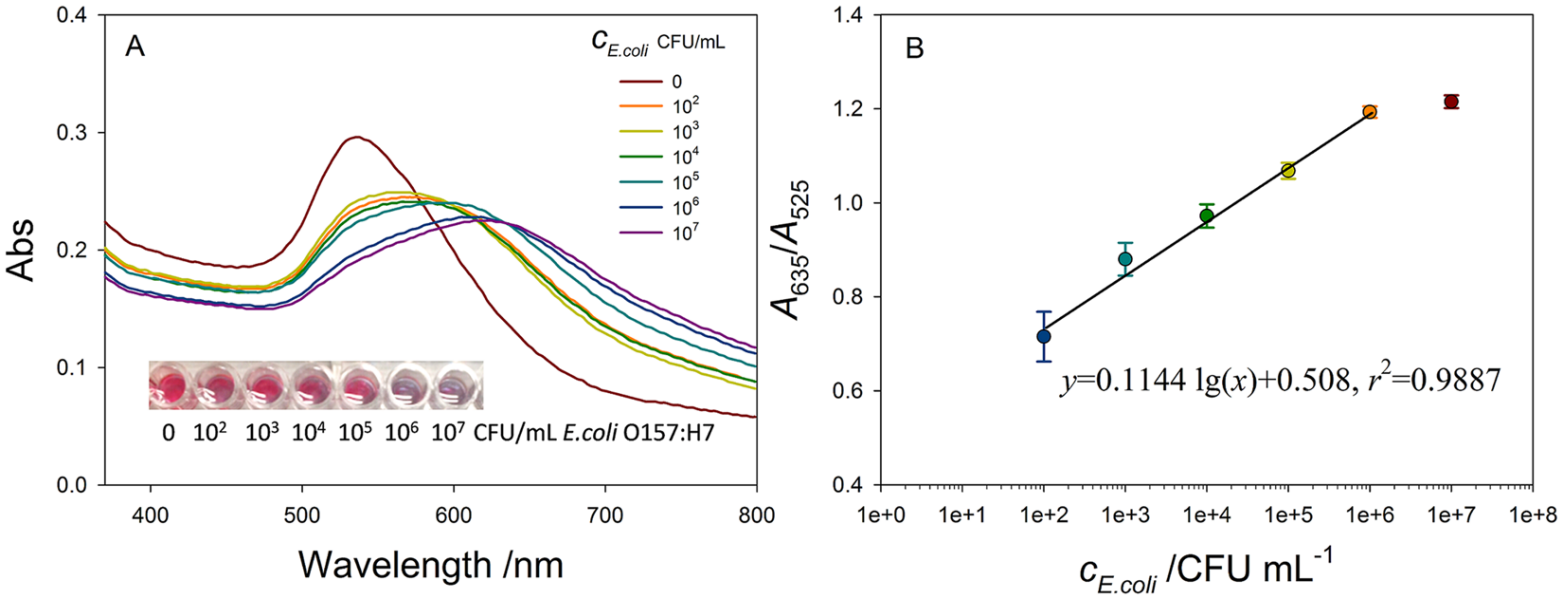
(**A**) UV-vis spectra of dextran@AuNPs with released Con A in different concentration of *E*. *coli* O157:H7. Inset is the photos of dextran@AuNPs mixed with released Con A at the concentration of 0, 10^2^, 10^3^, 10^4^, 10^5^, 10^6^, 10^7^ CFU/mL bacteria. (**B**) Linear relationship between the absorption change ratio *A*_635_/*A*_525_ and the concentration of *E*. *coli* (*N* = 3).

The UV-vis spectra for different concentrations of *E*. *coli* O157:H7 in the pure cultures were recorded (Fig. 5A), and the absorption ratio of *A*_635_/*A*_525_ was collected. It can be seen that the absorption in 525 nm decreases while the concentration of *E*. *coli* O157:H7 increases. Three parallel tests of *E*. *coli* O157:H7 at each concentration from 1 × 10^2^ to 1 × 10^7^ CFU/mL were conducted. We observed gradual color change from red to blue along with the increase of the concentration of *E*. *coli* O157:H7 (the inset photo in Fig. 5A). The ratio value of *A*_635_/*A*_525_ was plotted for different concentrations of *E*. *coli* O157:H7. The change in the normalized response was linear with the logarithm of *E*. *coli* concentration from 10^3^ to 10^6^ CFU/mL and had a regression equation of *A*_635_/*A*_525_ = 0.1144 log (*E*. *coli*) + 0.508, with a coefficient of determination *r* ^2^ = 0.9887. The limit of detection (LOD) was estimated to be 41 CFU/mL, which was calculated using the 3 *S*_D_/*m* equation, where *m* is the slope of the linear part of the calibration curve, and *S*_D_ is the standard deviation of the blank measurement. This LOD approximately agrees with the calculated value of the minimum number (2 to 393 bacteria cells) to induce the aggregation of dextran@AuNPs taking into account the sample volume is 0.2 mL and the effect of magnetic concentration. The LOD in our case is much lower than those reported in the literature using mercaptoethylamine-modified AuNPs for *E*. *coli* O157:H7 (from 2.91 × 10^8^ to 16 × 10^8^ CFU/mL)^41^. Also, the LOD using this method is comparable with that of colorimetric detection *E*. *coli* O157:H7 using Au@Pt nanoparticles as peroxidase mimetics^42^.

The total time for the test is about 95 min from bacteria capture to color change, which includes 45 min for EAbs-modified MNPs to capture bacteria, 5 min for washing and resuspending, and 30 min for binding of Con A, 10 min to change pH solution to obtain Final solution, and 5 min to detect UV-vis spectrum associated with color change. It is also very convenient to get the results, just by using as-prepared antibody-modified MNPs and dextran-modified AuNPs as the capture reagents, with a magnetic separator and some specific buffer solutions according to the operating procedures.

**Figure 6:**
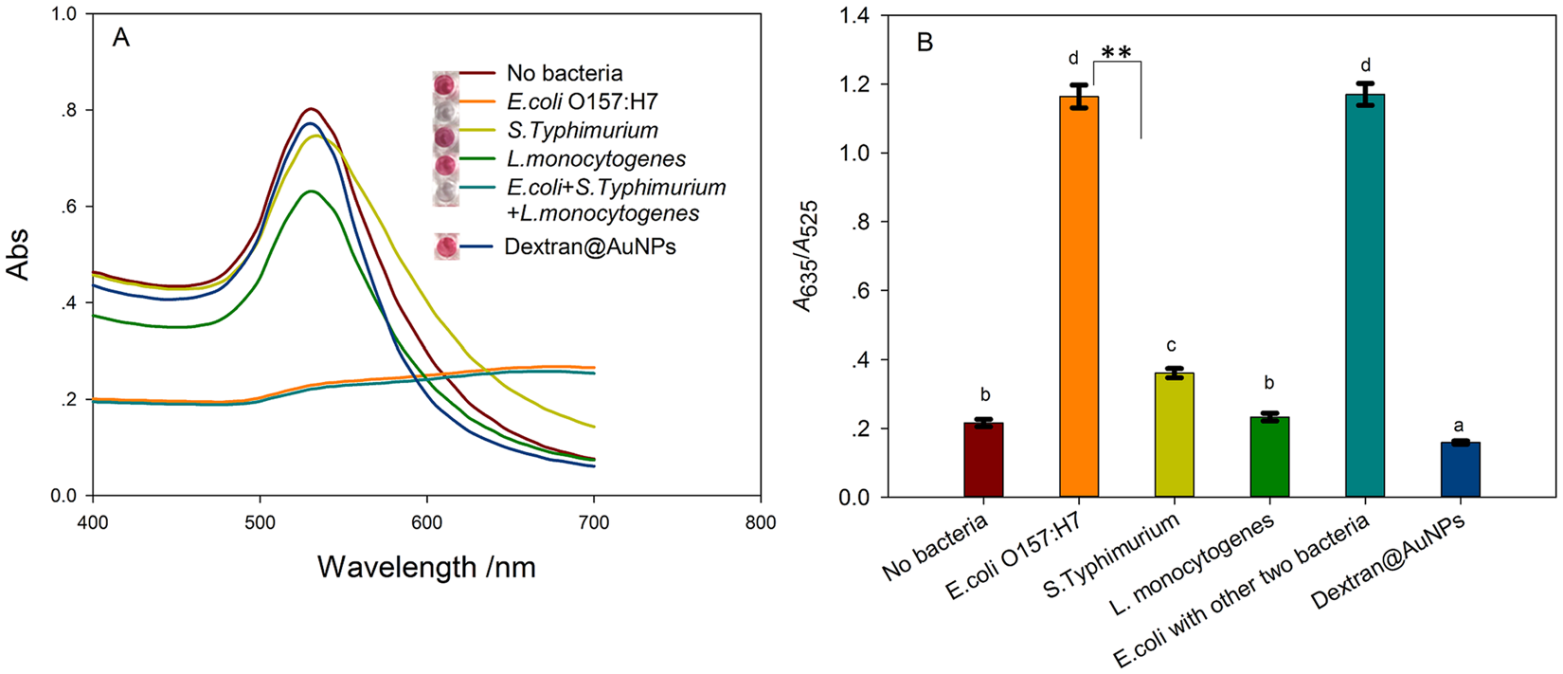
(**A**) UV-vis spectra of dextran@AuNPs with released Con A toward the same concentration (10^7^ CFU/mL) of *E*. *coli* O157:H7, *S*. *Typhimurium*, or *L*. *monocytogenes*. (**B**) Histogram of absorption ratio at 635 nm and 525 nm with different kind of pathogenic bacteria. Duncan’s multiple range test, alpha < 0.05; **p < 0.001.

The specificity of this colorimetric biosensor was investigated by using *S*. *Typhimurium* and *L*. *monocytogenes*, which are known to potentially present in dairy product. Samples of both individual *E*. *coli* O157:H7, *S*. *Typhimurium* and *L*. *monocytogenes* and their mixture were used. As shown in Fig. 6A, the response of *S*. *Typhimurium* and *L*. *monocytogenes* were all 30% lower than that of *E*. *coli* O157:H7. When in the presence and absence of *S*. *Typhimurium* and *L*. *monocytogenes*, the responses of *E*. *coli* O157:H7 kept the same. Statistical analyses using Duncan’s multiple range test was performed in Fig. 6B. No significant difference between *E*. *coli* O157:H7 and complexes with all three bacteria was observed, while there is a significant difference between *E*. *coli* O157:H7, *S*. *Typhimurium* and *S*. *Typhimurium*, with F = 1666.8, *p* < 0.0001. The difference in the median values in two groups between *E*. *coli* O157:H7 and S. Typhimurium is greater than would be expected by chance, i. e., there is a statistically significant difference (*p* < 0.001). The results signified that the colorimetric biosensor could be used to detect *E*. *coli* O157:H7 in the presence of other microorganisms. Therefore, the proposed strategy could reduce the risk of false positive determination caused by other microorganisms. The high specificity of the antibody and the magnetic separation procedures should contribute to the satisfactory specificity of the method.

In order to verify the feasibility of the method for practical analysis of *E*. *coli* O157:H7, the colorimetric biosensor was tested using fresh milk. Briefly, 1 mL of milk sample was diluted to 10 mL with PBS (pH 7.4), and then spiked with desired concentrations of 1.0 × 10^3^, 1.0 × 10^4^ and 1.0 × 10^5^ CFU/mL *E*. *coli* O157:H7. Subsequently, tests were performed using the proposed method. The EAbs modified MNPs suspension was incubated with milk sample for 45 min. After washing with PBS twice, the bacteria-bound MNPs were resuspended in PBS reaction solution containing 0.5 mg/mL Con A. The mixture reaction solutions were sequentially washed for three times to remove the unbound Con A. Then bacteria solutions were resuspended in pH 5 buffer to release Con A dimer, followed by transporting supernatants to new tubes and adjusting pH back to 7 by NaOH (termed as Final-solution). Finally, these F-solutions were mixed with 50 μL dextran@AuNPs for about 5 min, absorption spectra were collected using UV-vis spectrophotometer. Then the final results were obtained according to the calibration equation. As shown in Table 1, the recoveries of standard additions are in the range of 94–116%, indicating acceptable accuracy of the colorimetric biosensor. The results demonstrated that the proposed strategy can be applied to real samples and has the potential for practical applications.

**Table 1:** Recovery studies of spiked *E*. *coli* O157:H7 in fresh milk samples^a^.

added *E. coli* concentration (CFU/mL)	*A*_635_/*A*_525_	found (CFU/mL)	recovery (%)	RSD^b^ (%)
1.0 × 10^3^	0.850 ± 0.012	0.99 × 10^3^	99	1.4
1.0 × 10^4^	0.973 ± 0.008	1.16 × 10^4^	116	0.8
1.0 × 10^5^	1.076 ± 0.012	0.94 × 10^5^	94	1.1

^a^Average of three parallel tests. ^b^RSD represents relative standard deviation for absorption ratio.

## Conclusions

We have developed a facile and efficient colorimetric biosensor for the determination of *E*. *coli* O157:H7 using Con A. The Con A with strong affinity to the glycosyl sites could be labeled onto the surface of bacteria and induce the aggregation of dextran@AuNPs effectively. On the other hand, based on the interesting pH-dependent conformation-transformation behavior, Con A could be innovatively released from the bacteria surface and then be reconstructed based on the pH-regulation. Therefore, we realized sensitive colorimetric biosensing of large-sized (μm-scale) targets such as bacteria, which is generally not suitable for using of this kind of method. The biosensor exhibited a linear detection range from 1 × 10^2^–1 × 10^6^ CFU/mL (*R* ^2^ = 0.9887) and a calculated detection limit of 41 CFU/mL (*S*/*N* = 3), short assay time (~95 min), as well as satisfactory specificity. The biosensor also worked well for the detection in real milk sample and hence has the potential in practical applications. This method may provide a universal concept for the design of colorimetric biosensors for bacteria and virus. The pH-dependent conformation-transformation of Con A may also be used for design of other smart response device.

## Electronic supplementary material

Supporting Information
