# Supplementary material for: Exploiting pH-Regulated Dimer-Tetramer Transformation of Concanavalin A to Develop Colorimetric Biosensing of Bacteria
Source: Sci Rep. 2017 May 3;7:1452. doi: 10.1038/s41598-017-01371-6 (PMC5431225; doi:10.1038/s41598-017-01371-6)
Supplement: Supplementary file 1 — Supporting Information [file 41598_2017_1371_MOESM1_ESM.pdf]

Supporting Information

Exploiting pH-Regulated Dimer-Tetramer Transformation of Concanavalin A to Develop Colorimetric Biosensing of Bacteria

Xiahong Xu[†], Yuwei Yuan[†], Guixian Hu[†], Xiangyun Wang[†], Peipei Qi[†], Zhiwei Wang[†], Qiang Wang[†,*], Xinquan Wang[†,*], Yingchun Fu[‡], Yanbin Li[‡], Hua Yang[†]

[†] *State Key Lab Breeding Base for Zhejiang Sustainable Plant Pest Control; Ministry of Agriculture Key Lab for Pesticide Residue Detection; Key Laboratory of Detection for Pesticide Residues and Control of Zhejiang Province; Institute of Quality and Standard for Agro-products, Zhejiang Academy of Agricultural Sciences; Hangzhou 310021, China*

[‡] *College of Biosystems Engineering and Food Science, Zhejiang University, Hangzhou 310058, P.R. China*

[*] Corresponding author: Fax: +86 571 86401834; Tel: +86 571 86404355
E-mail address: qiangwang2003@sina.com

[*] Corresponding author: Fax: +86 571 86415202; Tel: +86 571 86419051
E-mail address: wangxinquan212@163.com

Table. S1. SEs of MNPs against *E. coli* O157:H7.

N_{pc} (cfu)	N_U (cfu)	N_{ms} (cfu)	SE (%)	Average SE (%)
	2	102	98.1	
$(1.04 \pm 0.05) \times 10^2$	2	102	98.1	98.2 ± 0.18
	3	101	98.5	
	21	999	97.9	
$(1.02 \pm 0.04) \times 10^3$	9	1011	99.1	97.6 ± 0.98
	42	978	95.9	
	99	10701	99.2	
$(1.08 \pm 0.07) \times 10^4$	504	10296	95.3	97.1 ± 1.49
	350	10450	96.8	

N_{pc} is the number of the positive control, N_{ms} is the number of the magnetically separated bacteria cells, N_U is the number of uncaptured cells (in supernatant and washed solution), SE is the separation efficiency.

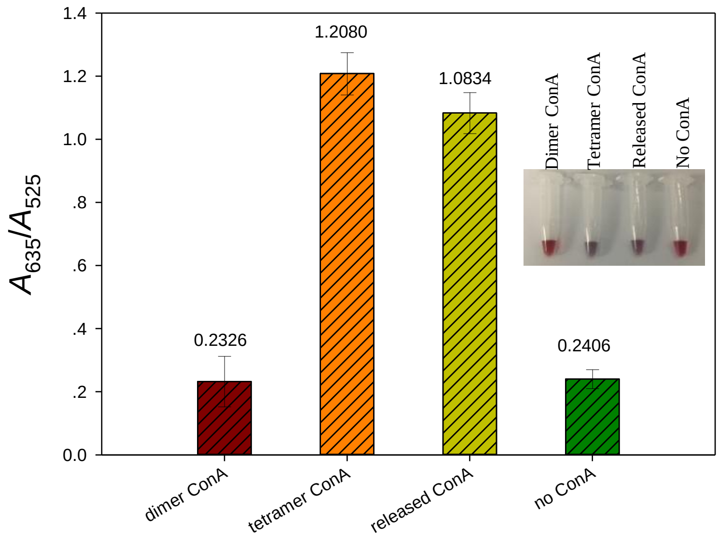

Figure S1. Histogram of absorption ratio at 635 nm and 525 nm based on mixed dextran@AuNPs with 6 μg/mL dimer ConA (pH 5), tetramer ConA (pH 7), released ConA (change pH 5 to 7 using 0.1 M NaOH), and the control without ConA.

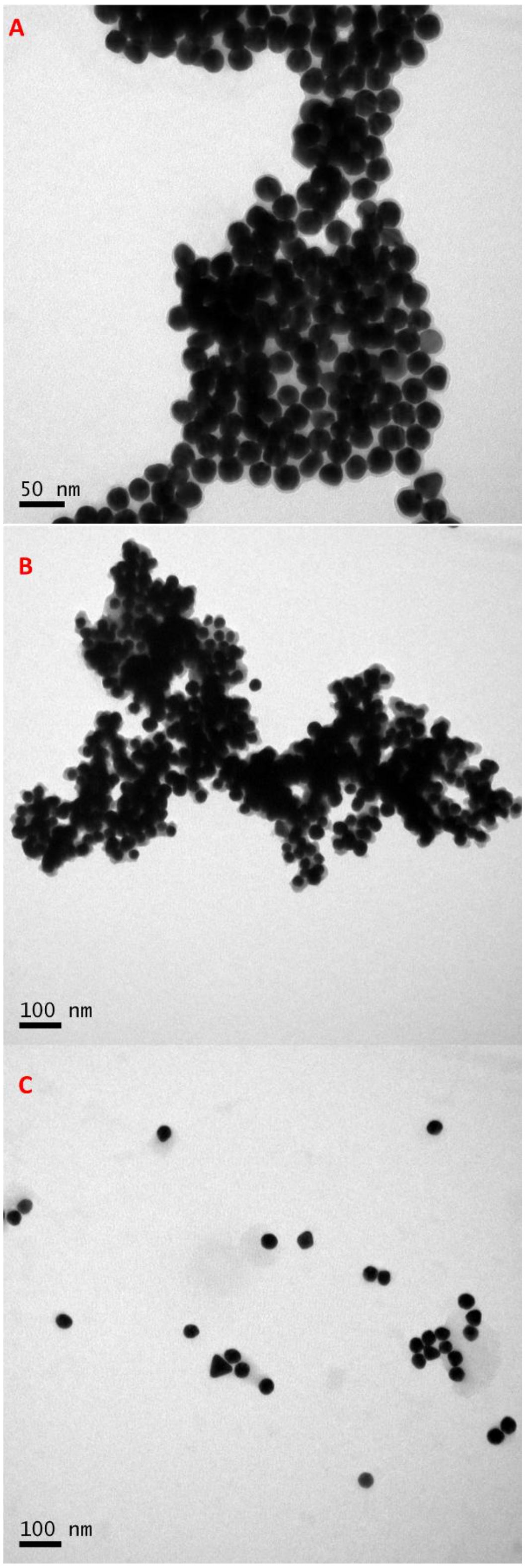

Figure S2. TEM imaging of dextran@AuNPs mixed with 8 μg/mL ConA (A), with released ConA in the F-solution (B), and with control in the absence of ConA in the F-solution (C).
